# Neurodevelopmental problems in pre-school children in rural Western Cape, South Africa: is community screening feasible?

**DOI:** 10.1186/s12888-025-06791-7

**Published:** 2025-04-08

**Authors:** Ben Truter, Christopher Gillberg, Amy L. Slogrove, Petra Conradie, Eva Billstedt, Lucy Thompson

**Affiliations:** 1 Gillberg Neuropsychiatry Centre, Sahlgrenska Academy, University of Gothenburg, Gothenburg, Sweden; 2 Department of Global Health, Faculty of Medicine & Health Sciences, Ukwanda Centre for Rural Health, Stellenbosch University, Cape Town, South Africa; 3 The Neurodiversity Centre, Paarl, South Africa; 4 Department of Paediatrics & Child Health, Faculty of Medicine & Health Sciences, Stellenbosch University, Stellenbosch, South Africa; 5 Institute of Applied Health Science, Centre for Health Science, University of Aberdeen, Inverness, UK

**Keywords:** Neurodevelopmental, Screening, Children, ESSENCE, South Africa

## Abstract

**Background:**

There is no standard public health screening for neurodevelopmental disorders in pre-school children in South Africa. There are pragmatic challenges in implementing such a programme in under-resourced regions. This study aimed to assess the feasibility of introducing a two-stage screening model for neurodevelopmental disorders among pre-school children in the rural Western Cape, South Africa.

**Methods:**

We adopted a mixed methods approach to evaluate the feasibility of using a brief screening questionnaire, the ESSENCE-Q, translated into local languages (Afrikaans and *isi*Xhosa), through verbal administration to mothers of pre-school children by trained research assistants without professional health qualifications within a cross-sectional ESSENCE-Q validation study. A focus group interview with research assistants who conducted the screening was analysed using reflexive thematic analysis. Feedback from participating mothers was obtained through a simple verbally administered questionnaire, rating 7 items on a Likert scale. Finally, the research team’s field notes were reviewed to critically assess feasibility.

**Results:**

The focus group interview identified areas of logistical challenge but highlighted positive reception among mothers. Since mothers generally had low levels of education and literacy, time was required to clarify certain child development concepts during screening. The in-person mode of engagement and flexibility of processes, including the use of a visual consent booklet, supported feasibility. Considerable resource limitations and trauma were encountered. Training key community members to administer the ESSENCE-Q for future studies or screening projects was considered feasible, provided neurodevelopmental training and trauma support is provided. Feedback from mothers was consistently positive, although unavoidable delays between assessment and feedback may have impacted this data. Identified challenges can be adequately addressed through practical adaptations.

**Discussion:**

Thorough preparation prior to commencing screening is considered essential for feasibility, including community-based stakeholder consultation, broad consultation around translated screening questionnaires, and preparation for diagnostic assessment. Ongoing support to mother–child recipients as well as those administering the screening, is required. The experience of participating mother–child pairs must be placed at the centre of community-based screening processes. Long term sustainability requires adequate training, supervision and psychological support for those administering such processes.

**Supplementary Information:**

The online version contains supplementary material available at 10.1186/s12888-025-06791-7.

## Introduction

The prevalence of neurodevelopmental disorders (NDDs) is not well established in Southern Africa. Reported international prevalence data vary considerably, depending on location as well as methodological and contextual factors such as underdiagnosis, and/or the state of knowledge and awareness of NDDs in a general population [1]. Prevalence studies have mostly tended to focus on singular NDDs, with far less focus on NDDs as a group. The overlap, co-occurrence, and heterogeneity of NDDs is now well-supported where there is data, with available European, Asian and North American estimates for NDDs as a group ranging from at least 10 percent [2] to 15 percent of all children [3]. A recent large-scale epidemiological study in Kenya found an overall adjusted prevalence of 90.8 per 10,000 [4]. The social- and educational burden of undiagnosed and unsupported NDDs is well established [5]. Yet, there is limited awareness and knowledge of NDDs in under-resourced rural areas in sub-Saharan Africa (sSA) and very limited access to specialised neurodevelopmental services [6].

Screening forms a key component of knowledge creation around NDDs. There is limited research on neurodevelopmental screening for pre-school children in sub-Saharan Africa (e.g. 7, 8). Screening questionnaires for NDDs have mostly been developed in high income (HI) countries and validated on HI populations using languages in the Indo-European group. There is a need for standardised, valid and reliable screening tools that can feasibly and economically be used in sSA in both colonial (Indo-European) language and African language groups [6].

A scoping review on research regarding NDD screening in young children in sSA [7] highlighted the need for simple, brief screening instruments that may be appropriately used in sSA to identify a broad range of NDDs. Previous studies used the Neurological Disorders Screening Tool (NDST) [8] and the Parent Evaluation of Developmental Status (PEDS) [9] to screen for a broad range of NDDs, as well as a specifically developed 23 Questions (23Q) questionnaire adapted from the WHO Ten Questions Questionnaire with added questions on autism [10].

The ESSENCE-Questionnaire (ESSENCE-Q) [11] is a brief but broad-ranging neurodevelopmental screening tool that could help fulfil this need. Based on the concept of ESSENCE (Early Symptomatic Syndromes Eliciting Neurodevelopment Clinical Examinations), it is intended to detect the need for further assessment in any of one or more specific developmental domains. Its validity has been demonstrated in Sweden [12], Japan [13] and India [14] but not yet on the African continent. Its advantages, where successfully used, include that it is quick and easy to administer and may detect children in need of further assessment for any type of NDD [13].

The acronym ESSENCE was coined by Professor Christopher Gillberg [15] to underscore the interconnected, interwoven nature of the symptoms of various neurodevelopmental disorders in early childhood**.** Different NDDs often exhibit shared, intertwined features and overlapping symptoms, particularly during early childhood development [2, 16]. Therefore, a comprehensive, holistic approach to neurodevelopmental challenges is needed, including screening for NDDs that encompass multiple dimensions.

Careful consideration should be given to ensuring community inclusion through structured consultation when preparing for NDD screening in resource-deprived, rural settings. Kakooza-Mwesige et al. [10] describe extensive processes of community consultation and involvement prior to rolling out a screening project to overcome potential barrier factors such as low levels of awareness of NDDs and possible social stigma. It is crucial that such context-specific challenges are appropriately identified and addressed, to ensure valid individual screenings as well as fully informed consent. We set out to investigate the ESSENCE-Q as a potentially feasible resource for contextually-sensitive, community-based screening in this community.

## Aim

The aim of this study was to assess the feasibility of a two-stage screening model using the ESSENCE-Q neurodevelopmental screening tool, translated into two local languages (Afrikaans and *isi*Xhosa), administered verbally by trained persons without professional health qualifications in a rural, under-resourced community in the Western Cape Province of South Africa, to screen for a range of early childhood neurodevelopmental concerns.

The study was cognisant of the need to identify pragmatic factors that needed to be addressed and solved in place, specific to community, culture and location, for screening and for comprehensive assessment after screening to be provided sustainably and at scale. These are described.

## Methods

This study required two main areas of methodology to be developed: That for the screening model, and that for the feasibility assessment.

### Methods: screening model

The screening model consisted of a preparation phase prior to actual screening (including community consultation, translation processes, training screen administrators, and planning for post-screening assessment and sustained support) as well as the implementation phase (including recruitment, informed consent and screening administration and conducting post-screening assessment and sustained support).

### Preparation phase

#### Community consultation

The study was performed in De Doorns in the Breede Valley sub-district of the Western Cape Province of South Africa. The majority of the population in De Doorns sample experience severe structural disadvantage with regards to socioeconomic factors and early adverse childhood events (ACEs). Most families living around this town reside in sprawling, dense, informal settlements next to the national highway running through the valley. These settlements are significantly under-serviced when compared to South Africa’s nationally sub-optimal levels of service.

According to 2022 National Census figures, the population of the Breede Valley was 212 682 [17]. The population of De Doorns is estimated to be approximately 11 280 or 5,3% of the Breede Valley sub-district. Routinely collected provincial hospital statistics indicated that the Breede Valley sub-district had 7 421 births between 01 January 2018 and 31 December 2019. Disaggregated birth data for De Doorns specifically was not available, but crude extrapolation suggests that approximately 394 births may have been from De Doorns.

**Fig. 1:**
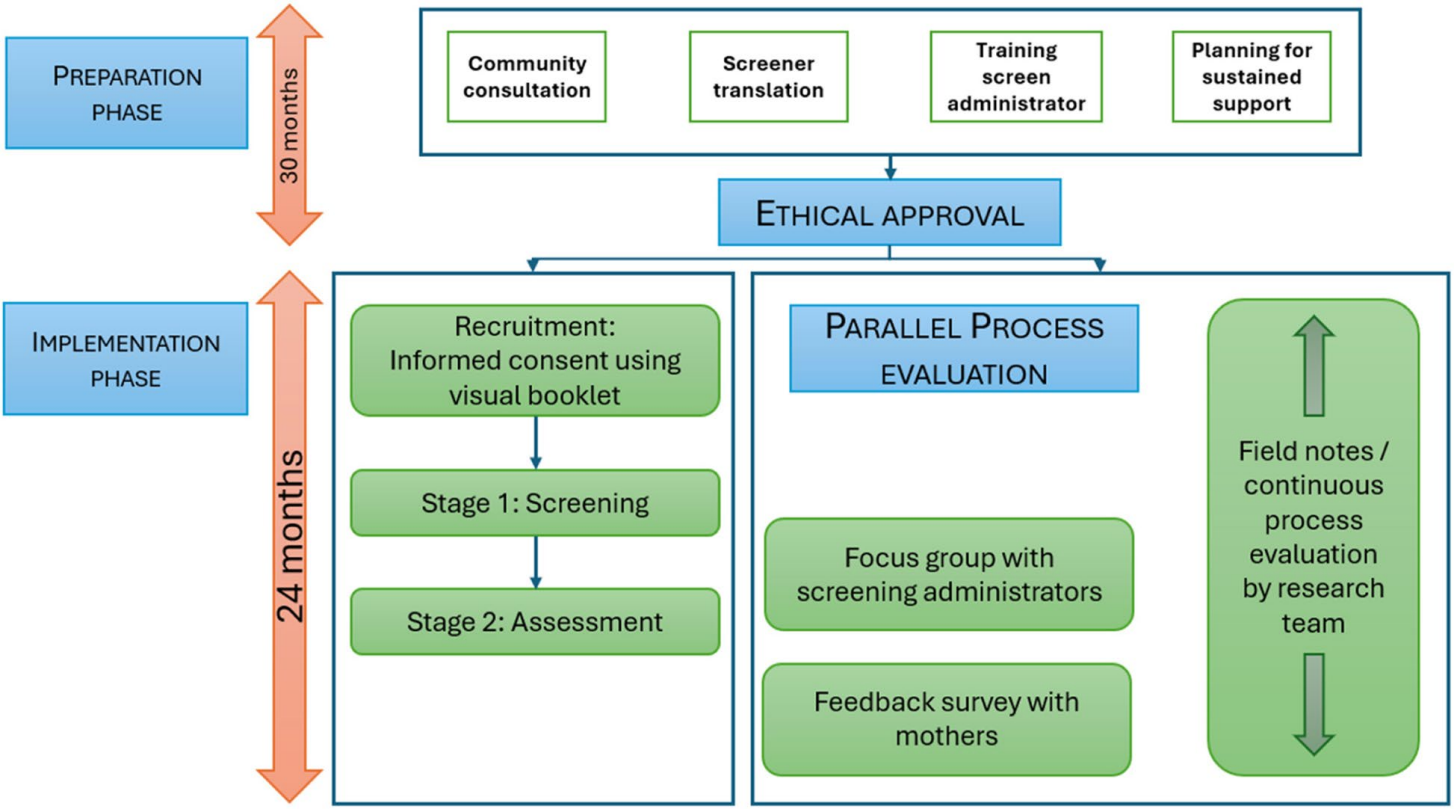
Flowchart of Study Phases: Preparation, Implementation and Feasibility Assessment

During the preparation phase 18 months prior to the onset of the study, the research team, consisting of the first author (a clinical psychologist), a community paediatrician, study coordinator, and research assistants (RAs), commenced initial engagement with interested stakeholders regarding this study and subsequent service provision. In this way, relationships were built well before commencing the study. Community consultation informed the development of the research protocol and ethics application. Stakeholders included staff working in the primary health care (PHC) clinic and large farming collectives in the area, as well as parents of older children with NDDs attending the local PHC clinic. Many families in the area are dependent on accessing early education for their child through the farming corporation(s) where parents/caregivers work. Local farming cooperatives that provide Early Childhood Development (ECD) facilities for their employees’ children were all contacted to ascertain their willingness to be part of the project. Significant interest was garnered as well as agreement in principle from five of the largest farming corporations (Fig. 1).

#### Screening instrument and translation

The ESSENCE-Q screening tool enquires whether caregivers have concerns regarding their child’s development in any of 12 listed areas (see Appendix A). Concerns are scored as Yes = 2, Maybe/a little = 1 or No = 0, yielding a summary score range of 0–24. A child is considered to screen ‘positive’ if a score of ≥ 3, or in some studies ≥ 4 [14], is recorded.

Afrikaans and *isi*Xhosa are the two most widely used languages in the Breede Valley sub-district where the study took place. The ESSENCE-Q, along with other screening tools used at a later stage of the broader study, were translated into the target language and then translated back. The Language Centre at Stellenbosch University completed the original forward and back translations of the *isi*Xhosa versions, and the Department of Afrikaans and Dutch conducted the Afrikaans translation. Care was taken to ensure that translators clearly understood the purpose of each item in the ESSENCE-Q and other screening questionnaires used in later stages of the study. Formal translations were reviewed through focus group discussions with RAs, who were very familiar with the colloquial language of the target community, as well as two focus group meetings facilitated by the first author (BT) with 9 parents of children with known NDDs at the local clinic. The purpose of these were to establish the most appropriate colloquial Afrikaans and *isi*Xhosa terminology and ensure that the precise concept behind each question was captured in the wording.

#### Training screening administrators

RAs with tertiary education but no professional health qualifications were trained to administer the ESSENCE-Q with mothers/guardians (henceforth referred to as ‘mothers’). Verbal administration was decided upon, due to low levels of education and literacy in the study population. RAs were selected based on their considerable familiarity with, and perceived acceptability to, the study population, as it was important that mothers felt comfortable with them. Training focused on reliably administering the ESSENCE-Q, as well as developing some understanding of early childhood development and NDDs. Training occurred across three days at the Stellenbosch University Worcester Campus. Day one comprised information about the study and its screens, as well as theory surrounding neurodevelopment in children from birth to 5 years, particularly the period 3—5 years. Training content was informed by consultation with a developmental paediatrician, a paediatric neurologist, a clinical psychologist, and a child and adolescent psychiatrist. Training prioritised markers of ‘typical’ development, in order to support trainees in spotting areas for concern. Videos and printed learning materials were used. Day two focused on technical skills such as adhering to ethical principles (e.g. confidentiality), data collection, data capturing, obtaining informed consent, non-guiding interviewing skills, lay-counselling and support skills (in the event of mothers experiencing distress requiring support), effective service referrals, and COVID- 19 safety protocol. Training was undertaken by a clinical psychologist (the first author), a developmental paediatrician and a paediatric neurologist, two of whom were present on any given day. Trainees (RAs) were given a simple printed summary of all information to take home and further familiarise themselves with. Trainees were tested on day three of training, two weeks later, about their understanding of the material and their confidence level in administering the screening tools. Training included discussion around their technical supervision as well as Stellenbosch University Human Resource procedures to access counselling services, should this be required as a result of any distressing experiences during recruitment and screening.

The first author and/or study coordinator had review sessions with the RAs fortnightly to ensure that any problems arising during the study could be addressed quickly and efficiently, mitigating challenges in further data collection. These sessions were also opportunities for debriefing (if/when required) and identifying possible need for counselling services.

#### Planning for sustained support

One of the established principles of screening programmes [18] is that diagnostic and intervention services should be available after screening. Though this was a research study, it was considered ethically imperative to follow this principle based on the necessity for accessible, embedded services for children included in the study. In the second stage of this study, not described in this paper, 100 of the children screened received further assessment in order to assess the validity of the ESSENCE-Q in this group. Due to the extremely limited availability of neurodevelopmental services in the research population, the research team initiated a project to deliver such services in a sustained manner through the creation and resourcing of an ‘ESSENCE’ outreach team. This included children who had been screened as part of this study, those who underwent assessment, as well as any other children in the community identified as requiring neurodevelopmental services. This team continues to work closely with existing governmental and non-governmental organisations to ensure embedded, long-term support.

### Implementation phase

#### Ethics approval

Ethics approval for the ESSENCE-Q validation study as well as the preceding feasibility activities was obtained from the Health Research Ethics Committee (HREC) of Stellenbosch University (S20/10/290 PhD). Further approval was obtained from the Western Cape Government Department of Health and Wellness to access the Primary Healthcare Clinic (PHC) in De Doorns (WC_202103_004). Informed and signed agreements to conduct the study were collected from private farm-affiliated early childhood development (ECD) centre owners. The study adhered to the Declaration of Helsinki regarding research carried out on humans, throughout all stages.

#### Recruitment and participant informed consent

A sample of mother–child pairs within the region were recruited from both PHC and ECD centre (creche) attendees on privately owned farms, by the RAs who were trained as screening administrators. Inclusion criteria were that the child participant had to have been born between 01 January 2018 and 31 December 2019 at one of the two primary birth sites in the region, either the Worcester Midwife Obstetric Unit or Worcester Provincial Hospital, to fall within the Breede Valley Maternal and Child Sentinel Site Cohort. All parents and guardians were eligible to participate, but only mothers and female guardians responded to recruitment.

The recruitment process started with the research team providing short information sessions about the study, repeated throughout the recruitment period, at the PHC and at ECD centres in the community. Mothers had the opportunity to register their interest in the study immediately afterwards, or to contact the RAs at a later stage (for example, through the contact details provided, through the ECD leader or through other community members). RAs continued to make themselves accessible in the community in various ways, such as being stationed outside the local PHC on child wellness clinic days, visiting the ECD centres, and moving around on foot in the community, informally telling people about the study. They wore clothing identifying them as being part of the study.

Mothers were invited to provide informed consent to initially answer only the short 12-item ESSENCE-Q. They could do so there and then, or arrange a suitable time and place to meet the RA again to conduct the informed consent-, enrolment- and ESSENCE-Q processes. These were conducted in settings selected by the mothers as being most comfortable: at home; indoors or outdoors on farms; at ECD settings on farms or in the community; at the local public health clinic; or at one of the local churches. A flexible approach was maintained in terms of rescheduling appointments, if families were unable to keep them.

The informed consent process and programme was presented both verbally and visually, in the form of a specially created Visual Consent Booklet. For mothers younger than 18 years, the guardian of the mother was required to sign an informed consent form, and the mother herself an informed assent form. Mothers had to be able to understand (in first language capacity) one of the three languages in which the study information forms and screens were provided (English, *isi*Xhosa or Afrikaans).

A small gift to the value of R50 (approximately USD2.50) was offered at recruitment to participants who signed the consent form and agreed to be part of the study.

#### Visual consent booklet

A detailed visual consent booklet explaining the study procedures, the ESSENCE-Q screen and all other information required to provide informed consent, was provided to each mother (Appendix B). This visual consent included all the components of the HREC approved full-text informed consent document, in illustrated form, and was approved by the Stellenbosch University HREC for stand-alone use. It contained the following sections:

- What is this booklet about and why are you being given this booklet?
- What is the research project about?
- Why do we want you and your child to take part in our project?
- Who will know you are in the study and how will we protect your data?
- What will be the benefits and risks of taking part in the study?
- Can my child or I get hurt in any way this study?
- What happens if you say no?
- What happens now if you say yes?
- Notes and contact details.

The use of a visual consent booklet was intended to ensure that consent could be fully and comprehensively informed, even if mothers possessed low levels of literacy and/or formal education. We also requested basic contact information and consent to being contacted for potential further participation in the study. Informed consent for this second detailed data collection phase was conducted in-person using the informed consent booklet at the point of initial screening, witnessed by a second screen administrator, and again verified verbally at the time of the follow-up in-person interview.

#### Screening administration

Following informed consent, all mothers were asked to answer the 12-item ESSENCE-Q. Administering the ESSENCE-Q took no more than ten minutes and was conducted verbally in the language of the informants. This took place during March 2022 to June 2023. Follow-up detailed assessments were made available subsequently for children who screened positive on the ESSENCE-Q. Additional follow-up and referral to appropriate community-based support services, primarily the ESSENCE outreach team headed by a Clinical Psychologist, was offered to all mother–child pairs where concerns were identified. Consent procedure included requesting permission for the outreach team to initiate communication with caregivers to support access to intervention services after the study.

Study data was collected and managed using the Research Electronic Data Capture (REDCap) tool [19]. REDCap is a secure, web-based software platform to support data capture for research studies. During the study period, all screening and consent data was immediately anonymised and entered into the REDCap database hosted at Stellenbosch University, using portable devices. A designated data coordinator who is a staff member of Stellenbosch University, managed REDCap data access. Informed consent forms were the only paper data records and were securely filed in a locked cabinet in locked storage rooms on site.

### Methods: assessing feasibility

Extrapolating from the regional numbers of births, we assumed a rough estimate of approximately 394 live births in De Doorns during the study cohort period. We intended to screen all or as many of these children as possible and estimated that doing so would result in approximately 50 children screening positive on the ESSENCE-Q (sufficient for further statistical analysis). This was based on extrapolation from international data on the prevalence of ESSENCE problems, estimated as approximately 10% in countries where it has been investigated [13, 20], along with our expectation of a somewhat higher prevalence in our sample due to a variety of adverse exposures and resource deprivation. We anticipated that it would be feasible to recruit approximately 394 mother–child pairs in one year with 2–3 RAs recruiting 4 days a week.

Feasibility of this screening model was further assessed along the following dimensions using mixed methodologies (parameters to determine feasibility described in parentheses):

- Experience and feedback of screening administrators through focus groups and qualitative thematic analysis (predominantly positive endorsement of the instrument, the process of screening, and administrators’ ability to manage additional questions or problems arising during contact with the participants, would support feasibility);
- Experience and feedback of mothers through short, verbally administered feedback questionnaires (80% or more positive responses would support feasibility);
- Research team’s field notes and critical reflection on factors to be considered when undertaking such screening (feasibility would be supported if problem areas identified by mothers, administrators or the research team could be resolved through relatively simple adjustments without changing the fundamental focus of the project);
- Recording the time, resources and costs involved in training screen administrators, as well as providing any required ongoing support (resource requirements would be recorded only as a guide for future replication of this model).

### Research assistants’ feedback and experience

A focus group interview was conducted with the three RAs who were trained to administer the screening tool, by an independent member of the research team who was not involved in data collection. Open-ended enquiry was made as to their experience of the recruitment process, challenges encountered, general reception from participating mothers, relevance of the instrument to the community, and perceived replicability of this model. While loosely adhering to these broad categories of enquiry, discussion was guided by what appeared to be meaningful to the participants. Care was taken not to lead the conversation in any pre-defined direction but for any questions to build on the content shared by the RAs. The interview was transcribed in full. Reflexive thematic analysis [21] was conducted. Following familiarisation with the data, initial codes were independently generated by two researchers and reviewed by the first author. Preliminary themes were generated through reflexive collaborative discussion and then refined further. Themes were assembled and reviewed for quality, coherence and boundaries. Final themes were defined, named and reported. A constructionist epistemology was adhered to, along with an experiential orientation. A predominantly inductive approach was followed [22]. Both semantic and latent coding were used in our analysis.

### Mothers’ rating of their experience

A simple feedback questionnaire consisting of 7 statements and a 5-point Likert rating scale (Table 1), was created by the research team for mothers to rate their experience of their interaction with the RAs, the ESSENCE-Q and the relevance of the ESSENCE-Q to their lives and community. The 5-point Likert scale consisted of the options: *1) Do not agree at all; 2) Do not agree; 3) Uncertain; 4) Agree; 5) Strongly agree*. Ratings of 4 and 5 denoted positive experiences, whilst ratings of 1 and 2 denoted negative experiences. Simple colour pictures ranging from unhappy to smiling faces accompanied each description for visual support.

**Table 1:** Summary of Mother’s Feedback for each Item on Rating Scale (n = 91)

ITEM	1	2	3	4	5	TOTAL
1. The research assistant was friendly and professional	0	0	0	6	85	91
2. I felt comfortable with the research assistant	0	0	0	9	82	91
3. The assistant could explain to me what the questionnaire was about in language I could easily understand	0	0	0	6	85	91
4. The assistant explained to me what neurodevelopmental problems in children are and what signs to look for, in words that I could understand	0	0	0	14	77	91
5. I will recommend this service to other parents in my community	0	0	0	12	79	91
6. It will be practical and easy to use the ESSENCE-Q in my community	0	0	0	12	78	90^a^
7. Completing the questionnaire taught me more about my child’s development	0	0	0	10	81	91
TOTAL NUMBER OF RESPONSES	0	0	0	69	567	636

^a^1 missing value

It was not practically feasible to reach all 153 enrolled mothers that participated in screening, to obtain feedback. Only the mothers of 100 children that underwent further assessment could be approached to complete this questionnaire during follow-up with the research team. Questionnaires were completed by 91 mothers between 8 and 22 months after the ESSENCE-Qs were administered (depending on when mothers completed the ESSENCE-Q). Only the Afrikaans translation of the feedback questionnaire was used, as all participating mothers chose to communicate in Afrikaans (Appendix C).

Mothers completed the feedback questionnaires when they met with the outreach team psychologist for follow-up support. This was done in small groups in the waiting area. The psychologist explained confidentiality and informed consent whilst emphasising that the questionnaire referred to the ESSENCE-Q specifically (and not to any subsequent assessment). Mothers were reminded of what the ESSENCE-Q questions were, who administered it with them, and where. The psychologist then read through the statements with the mothers item-by-item, and ensured they understood how to complete the questionnaire.

The proportion of mothers who positively endorsed any given item, was calculated, as well as the total number of mothers whose item-selections scored the study procedures ≥ 80%.

### Research team fieldnotes

Information obtained during the focus group interview was supplemented by qualitative feedback from the RAs and research coordinator on an ongoing basis throughout the period of data collection, as well as the researchers’ critical reflection on the study processes.

### Resource implications

Procedures regarding training sessions were recorded for future replication and analysis of time and resources needed to conduct such training.

## Results

Recruitment was slower than anticipated due to limited staff resources. RAs were available for recruitment only two days per week, rather than four as originally intended, due to unplanned involvement in other studies. No more than two RAs were available per day. RAs recruited and screened 185 participants over a 12-month period, of whom only 153 were eligible for the study based on date of birth. This was, however, sufficient to reach the target of 50 children who screened positive. It also suggests that our original target of screening ± 394 children, may have been achievable with the intended amount of staff resources.

### Focus group interview

The main themes constructed through Reflexive Thematic Analysis were:

- Mode of Engagement
- Flexibility of processes/Informal, creative solutions
- Concept Accessibility
- Trauma and Resource Deprivation
- Participant Experience

Mode of engagement (style of interpersonal communication and the value of relationship): The value of in-person engagement was emphasised. RAs explained how they were able to take the time to explain concepts, provide examples, and answer mothers’ questions. During the screening itself, the opportunity to explain concepts to mothers in person was considered very significant. Providing those explanations in a respectful, non-judgemental manner was considered important. RAs sometimes walked around in the community to informally access mothers.

*“Letting the moms know that they are important, their kids are important, we’re not leaving them after the detailed assessments”*

*“It is a safe space to tell us any concerns that they have”*

Flexibility of processes (the degree of rigour in process flow, location and timing): Informal, flexible approaches to processes such as recruitment and making follow-up arrangements were identified by RAs as having been essential to successful outcomes in this study. Being available in the places frequented by mothers and young children was identified as a strength of the approach, particularly as trying to reach mothers by phone was fraught with challenges. Formal community resources, such as the PHC, also presented some obstacles, e.g. having a designated day of the week for mothers with young children to attend, thus impacting on recruitment. RAs therefore resorted to informal resources such as word-of-mouth communication and informal outreach in the community (e.g. setting up a table outside the clinic) to better access participants. They also made themselves available on Saturdays in order to reach mothers who worked full time.

*“I have found more people outside the clinic in the streets recruiting there than inside the clinic. If we have a slow day inside the clinic, then I ask E. if I can just drive around and walk around outside the clinic, then we can just see what I can do.”*

*“When you go to the clinic there are lots of people, so the nurses can’t tend to listen to everybody’s complaints, so you maybe go for an injection, you get the injection, and you must leave.”*

Concept accessibility (of the ESSENCE-Q and child development): RAs reported that the general level of formal education amongst the group of mothers was low and that their knowledge and awareness of child development varied. RAs had to explain some concepts to mothers; they noted that this was not difficult but required time to provide clarifying information. They found it useful to relate concepts to things mothers were already familiar with, such as behaviour of an older sibling, or other aspects of the child’s behaviour.

*“When I explain the ESSENCE-Q, I try to relate the example to what the mom might know, what is familiar to the mom. I think at the first one, there is this question about learning new things. So, I would relate that to the mom and ask ‘mom, you know when the child was grabbing things …’ I would try and give examples. Then the mom would say ‘oh you mean this’, then I can give another example till they understand. We need to try and relate it to their everyday experiences.”*

RAs reported that mothers told them that the visual booklet was particularly helpful and that they would refer back to it at home. RAs were strongly supportive of the possibility that others in the community could be trained to administer the ESSENCE-Q in the same way they did – for example ECD teachers, community leaders and nursing assistants. They felt that a basic level of knowledge of NDDs is important, and that some training prior to commencing screening administration would be required.

Trauma and Resource Deprivation (as experienced by the mothers in their lives and as secondary trauma experienced by the RAs): High levels of exposure to trauma, violence and poverty-based vulnerability was a significant theme and appear to be embedded in the study community. Once mothers were asked about their children’s development, information about traumatic experiences also emerged.

*“Because the moms have nobody else to talk to, so once they start talking to us, they tell us everything under the sun […] As soon as she left [...] I started crying[...] The things that they tell us are horrible".*

Limited psychosocial support in the community was apparent, both for dealing with severe trauma and for neurodevelopmental concerns.

“*They don’t know where to go if the child has a concern, they don’t know who to speak to, because we learn that once we ask moms something about their kids, they start talking about everything. I think moms don’t have that outlet to talk to someone”.*

As a subtheme, the need for screening administrators to have access to psychosocial support was also identified, to support any secondary trauma experienced. Though RAs were trained to provide some containment and support during such disclosures, their own feelings of helplessness and powerlessness when hearing about families’ trauma were apparent. All reported occasions where they felt overwhelmed by what they had heard.

Participant Experience: RAs spoke about the positive feedback from participating mothers as well as other community members, such as a school headmaster, who strongly supported the need for neurodevelopmental support. Several informal requests from mothers outside of the study catchment area were received, for similar support. One RA described:

*“Most moms ask: ‘Why are you only coming now?’, ‘My older child is already sixteen, why are you only coming now?’ ”.*

Only one mother was encountered who didn’t want to participate further due to her reported distrust of state services.

Another aspect of participants experiences was that of conflicting priorities. Getting time off work was difficult. The recruitment and screening processes therefore needed to be directed by, and implemented according to, the needs and priorities of the participants.

### Mothers’ experience

Ninety-one of the 100 mothers whose children had further diagnostic assessments as part of the ESSENCE-Q study, completed feedback questionnaires. Only ratings of 4 and 5 were given on all of the 7 items on the feedback questionnaires; thus 100% of responses were positive. Responses of ‘4’ (‘Agree’) were given in 10.8% of cases and responses of ‘5’ (‘Definitely Agree’) in 89.2% of cases. There was one missing value on Item 6. Additional descriptive comments provided by some of the mothers at the end of the feedback form, were all positive as well.

### Research team’s fieldnotes and observations

Various procedural challenges were encountered, for which adaptations were required during the course of preparing and implementing screening.

#### Telephone interviews

It was initially intended to use telephone interviews for data collection on the background questionnaire due to COVID-related restrictions during the planning phase of the study. Mock trials of this questionnaire found it to be too time-consuming for telephonic administration. Mothers furthermore seemed to prefer in-person contact. Many did not possess their own cell phones, have reliable access to electricity to charge their phone, or funds to use their phone, and internet coverage through the area was mixed. Some mothers provided the contact number of a neighbour, family member, colleague, or their place of employment. It was therefore problematic to reach them outside of normal working hours, and their privacy during telephone calls could not be ensured. Multiple calls were required to arrange a time for the actual telephone interview. Where mothers relied on using someone else’s cell phone, the owner required reimbursement for the use of their phone and battery power. Phone numbers frequently changed, some phones were lost or stolen, and due to unreliable/lack of electricity supply phone batteries were often uncharged. The intended use of telephone interviews was therefore discarded. This extended some sub-phases of the research plan and created long tails so that we could not always complete all actions for all mothers in the research sample before starting on the next. This overlapping of events caused significant delays to execution and finish dates of some activities.

#### Non-NDD concerns raised

Additional concerns raised by mothers during the screening process included: Physical or other abuse; exposure to domestic violence; substance- and alcohol-related problems in the household; disclosure of specific trauma-related events; accessing help for siblings or other family members; mental health concerns in mother or other family members; health- and feeding problems; requests for help to understand medical- and related information given to a mother during a clinic or hospital visit. These matters were recorded and reported to the study coordinator who arranged clinic or social support referrals as required, as well as to the ‘ESSENCE’ outreach team. Follow-up of each problem raised was expedited within the outreach team or through referral to the relevant collaborating state- and non-governmental services in the region, with whom relationships were established previously.

#### Contextually pragmatic factors for further assessment

Though the focus of the present study is on the feasibility of a screening model, we considered it ethically imperative to plan for follow-up assessment and intervention prior to the commencement of the study. Logistical challenges in this regard included optimal use of limited resources such as parents’ time, travel options and clinical personnel. Attempting to conduct some facets telephonically was considered but found to be unfeasible. Doing so in person, in advance of the assessment with the child, would require additional time away from work for mothers and additional travelling costs. All aspects of the detailed assessments therefore had to take place in person on the same day. A dedicated study vehicle and driver was needed to provide transport for mothers and children to and from their homes. A maximum of two detailed assessments per day were planned to allow sufficient assessment time should unforeseen delays occur in meeting mothers and children at their homes.

### Resource implications

A 3-day training for RAs without professional health qualifications was found to be satisfactory for the purpose. Training content, once developed, constitutes a reusable resource that may be rolled out across various settings with only minor adaptations if/when required, with some additional content to be considered. To deliver such training, clinicians experienced in paediatric neurodevelopment and/or child neuropsychiatry were required. This included suitably experienced psychologists, developmental paediatricians, paediatric neurologists, child- and adolescent psychiatrists. We found it beneficial to use two trainers from different disciplines for all 3 days to ensure comprehensive consideration of all dimensions of neurodevelopmental difficulty in young children. Regular supervision or monitoring sessions, ideally once a week or fortnightly for 30–60 min, were considered essential in order to support the RAs involved in screening. In our study this was provided by a psychologist. Accessibility of such support in the event of unexpected situations arising during screening contacts, is equally important – including opportunity for RAs to request and access support for any associated trauma experienced in dealing with the harsh realities of this community setting. The resource demand in this regard was more than originally estimated and planned for.

Consent procedure, answering questions about the study and responding to additional concerns about the child, the child’s siblings or other difficulties took approximately 30–60 min per participant. Screening itself using the ESSENCE-Q took at most 10 min per participant. However, establishing and maintaining contact with mothers to make practical arrangements for screening (and later, assessments) to be conducted, was time-consuming and often difficult. As described above, recruitment and screening were slowed by limited availability of RAs to conduct recruitment and screening and difficulties in contacting and then re-contacting participants. RAs also reported several requests for participation in the study from mothers living outside of the identified geographical area. Screening and assessment services were arranged (and provided through the outreach service) to ensure all requests were positively attended to, albeit not included for study purposes.

## Discussion

Using the proposed model of screening for a broad range of NDDs in young children in an under-resourced, rural part of South Africa can be feasible, provided that certain conditions are met. It was evident that thorough preparation prior to commencing screening was essential to ensure community understanding, acceptance and willingness to participate. Such preparation included community-based stakeholder consultation, discussion groups around perceived challenges and opportunities, broad and repeated consultation around translation of questionnaires in terms of local applicability, and planning for further clinical/diagnostic assessment and ongoing support.

Contextual challenges in the current study involved language, distance, lack of access to transport and difficulties in relation to communicating arrangements with participating mothers. Aside from the considerable vulnerabilities already experienced throughout this community, the limited social justice rights of participants—limiting their ability to seek, obtain, and follow sources of support for their child – affect how such a model can be successfully applied. Adaptations to standard procedures were required, tailored to the needs of the relevant setting, such as verbal screening administration and ensuring that participants experience comfort and some degree of identification with those administering the screening. Without such practical, logistical considerations and context-specific adaptations it is considered unlikely that the study would have been feasible.

The high levels of trauma encountered in the community necessitates an adequate framework of further support, both for caregivers themselves and for individuals administering the screening to caregivers. Future screening projects must be embedded in a network of supports allowing for onward referrals, which may be unique to each specific context. Further research is also required into the potential complex interactions between NDDs, trauma and socio-economic marginalisation in communities such as this, at the levels of identification, conceptualisation and support.

Recruitment was slower than anticipated, most likely due to resource restrictions, rather than lack of interest from mothers. Increased personnel capacity may have allowed for a greater proportion of the children to be screened. This resource-dependency and its demonstrable impact on our scope and reach, became clear early in the implementation phase, and will require an innovative response in related future projects. Nevertheless, the targeted number of children that screened positive was reached after fewer screenings than anticipated. This may reflect actual higher prevalence, and/or the high need for neurodevelopmental services in this area. Mothers with existing concerns about their child’s development may have been more motivated to sign up for the study in order to access support. The fact that only mothers and female carers were recruited, may limit the generalisability of the findings to fathers and male carers. However, this reflects the reality as reported during the community consultation phase, that care of young children in this community is predominantly carried out by women.

A strength of this study was ensuring the integrated, sustainable provision of ongoing support services through our community outreach team, after screening and assessment. A further strength was its focus on the experience of RAs as central to both study phases. Through their acceptability to the local community and their insights into how language and procedures were received by participants, they were able to identify possible or arising pragmatic concerns. Their sustained presence in the community appears to have been of importance, in terms of trusted relationship as well as to facilitate communication in the absence of reliable telephonic infrastructure. While their role as part of the research team may have motivated RAs to provide more positive responses in the interview stage, balance was sought by specifically enquiring about challenges, and by using an independent interviewer that had not been directly involved in the project and was not previously known to the RAs.

In light of previous findings that the reliability and validity of the ESSENCE-Q can be impacted by its being administered through direct interview [23], RAs were specifically trained to administer the screen focusing only on items as they are worded, thus not impacting responses. A balance needed to be struck, given that mothers might not know how to objectively rate their child’s developmental trajectory [13]. RAs therefore had to be familiar with typical development and be able to give adequate examples without biasing responses.

Using persons without professional health qualifications as screen administrators has been noted to be helpful in the administration of screening questionnaires in similar settings in South Africa [24], provided they are trained to demonstrate a non-judgemental outlook and are able to clarify what the core of screen items are referring to. Screening administrators may contribute to parents’ understanding of ESSENCE conditions, their awareness of available services, and prepare them for further contact and diagnostic assessment. Significant aspects of the typical referral chain may thus be transferred to future screening administrators, such as existing community- or primary health care workers in the public service system, in under-resourced South African settings.

Potential shortcomings of this study include that the sample may be biased towards mothers with existing concerns about their children, who have been more motivated to participate in the study. They may nonetheless represent the group most urgently requiring screening- and support services, which renders their support and ‘buy-in’ relevant in assessing the feasibility of this model. The feedback questionnaire presented several significant methodological shortcomings, included use of positively worded statements only; the extended period between screening and feedback (due to various unforeseen practical challenges); and the fact that it was only completed by mothers who participated in further assessment because of challenges in accessing all mothers. This may have biased the findings considerably. Additionally, mothers may have been reluctant to report dissatisfaction due to relational power imbalances; and/or their responses may have reflected their positive experience with follow-up support services (despite clear explanation that the feedback questionnaire pertained to screening). While it is acknowledged that the feedback questionnaire outcomes may therefore have limited validity, it does provide cautious qualitative support for the mothers’ positive experience of our screening model in this community. Further qualitative studies should employ interviews with mothers in order to obtain richer and more nuanced feedback of their views and experiences.

The study raised important questions about the impact of socio-economic context on the practicality of research methods; the realistic scope of comparable feasibility criteria; and the role of trauma in the screening process in resource-deprived communities. Many of these remain unanswered. However, it is apparent that in communities with high levels of compounded trauma (both through multiple specific events and sustained, persistent living conditions), adaptations to the screening process are required, along with extensive community consultation and trauma counselling where required.

Incorporating a model of screening through appropriate capacity-building with current community-based workers in both PHC and ECD settings presents a potentially feasible model for identifying children with possible NDDs, and presents opportunities for community-embedded, holistic intervention and training models.

## Supplementary Information

Supplementary Material 1.

## Data Availability

The datasets generated and/or analysed during the current study are not publicly available for ethical reasons, due to the sensitive and very personal nature of the qualitative data collected and the possibility of individuals being identified through the data, but are available from the corresponding author on reasonable request.
