# Supplementary material for: Neurodevelopmental problems in pre-school children in rural Western Cape, South Africa: is community screening feasible?
Source: BMC Psychiatry. 2025 Apr 8;25:348. doi: 10.1186/s12888-025-06791-7 (PMC11980325; doi:10.1186/s12888-025-06791-7)

**APPENDIX A**

**THE ESSENCE-Q SCREENING INSTRUMENT**


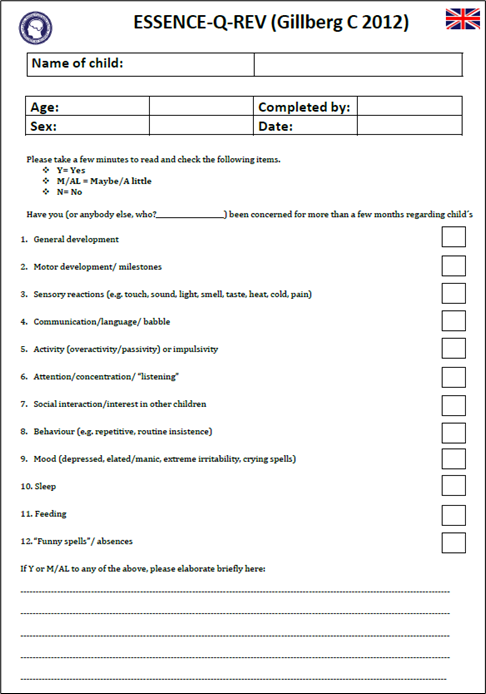


**APPENDIX B

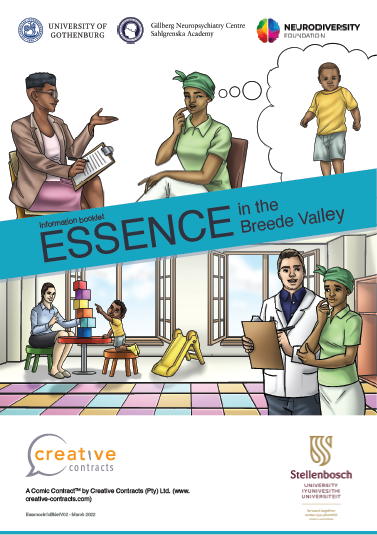

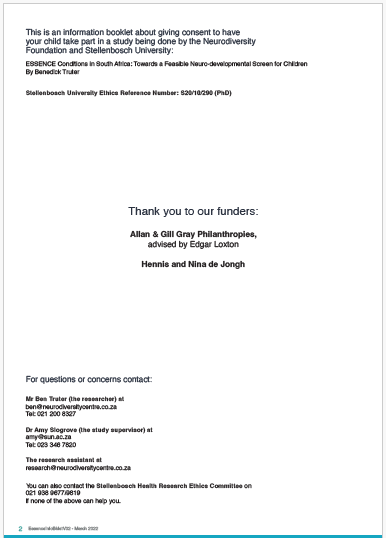
**

**
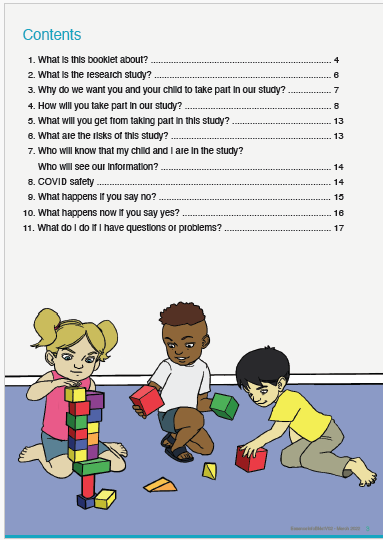

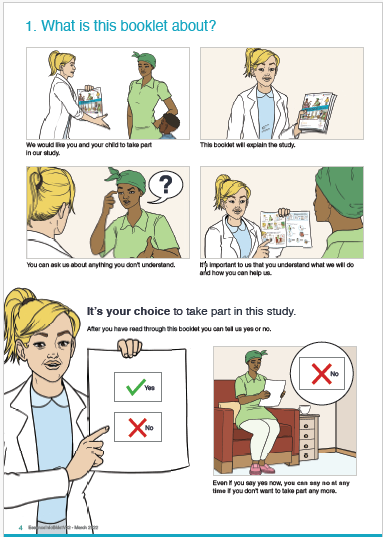
**

**
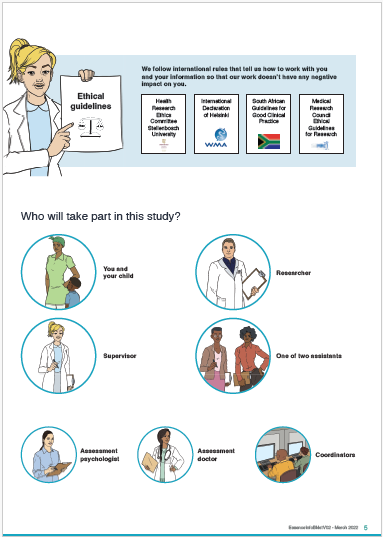

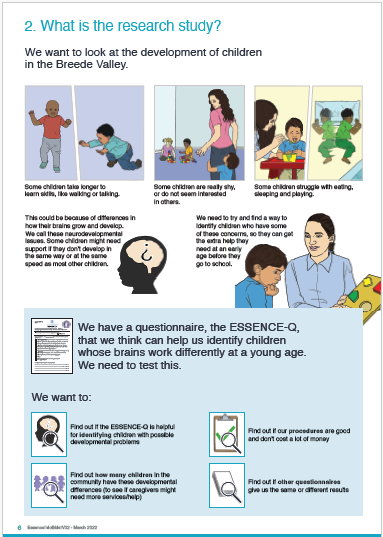
**

**
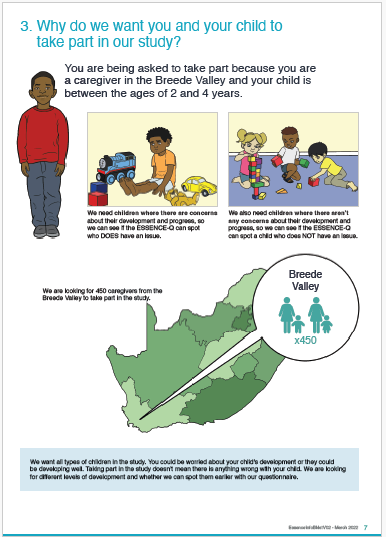

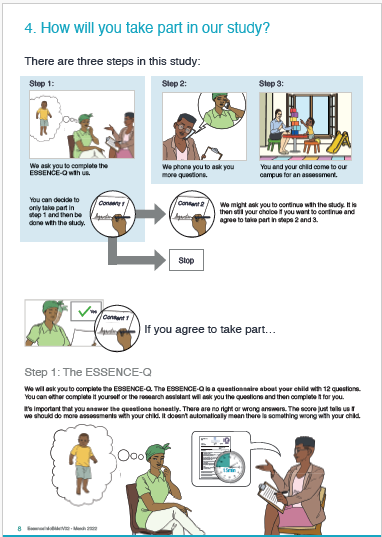

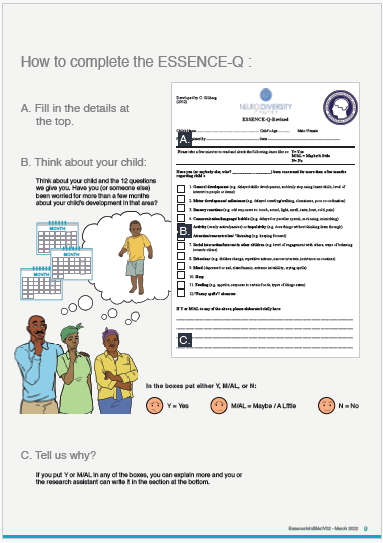

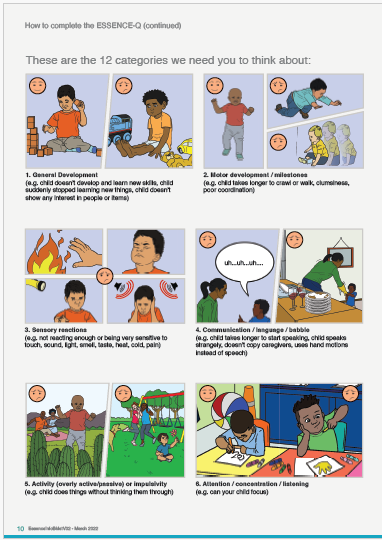
**

**
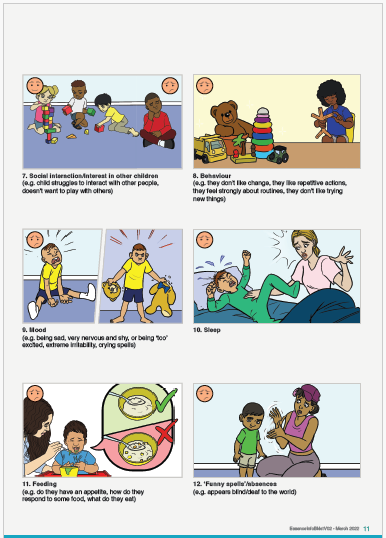

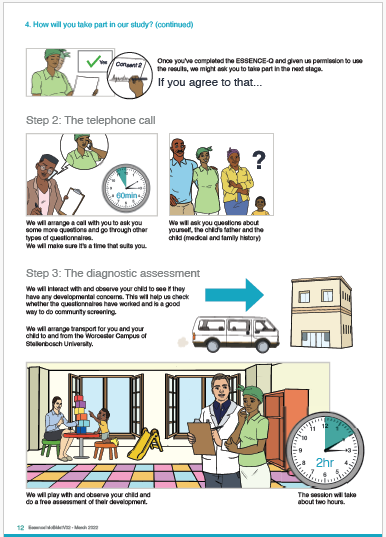
**

**
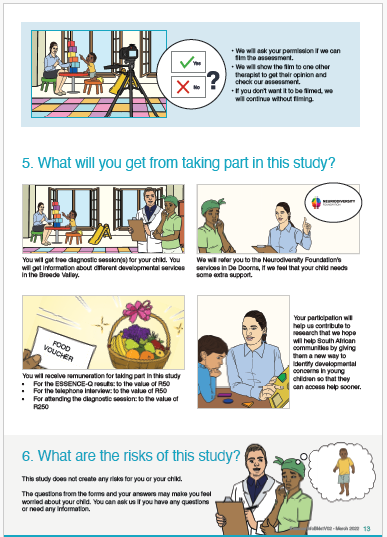
** **
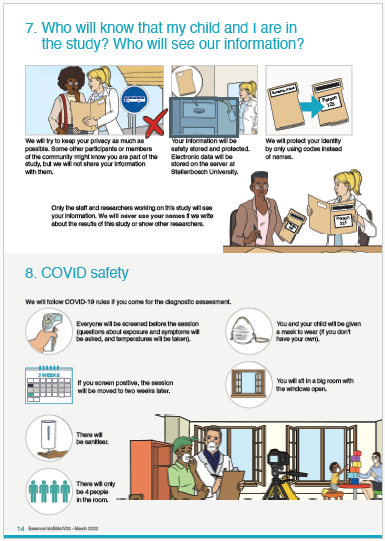
**

**
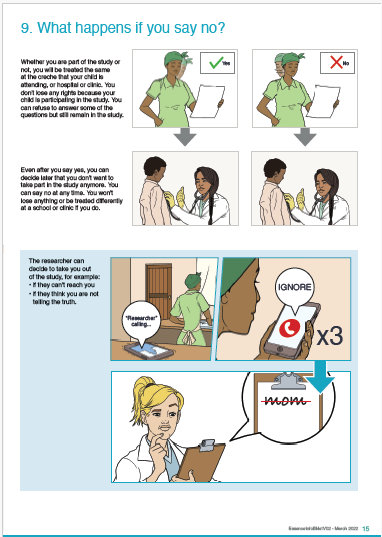
** **
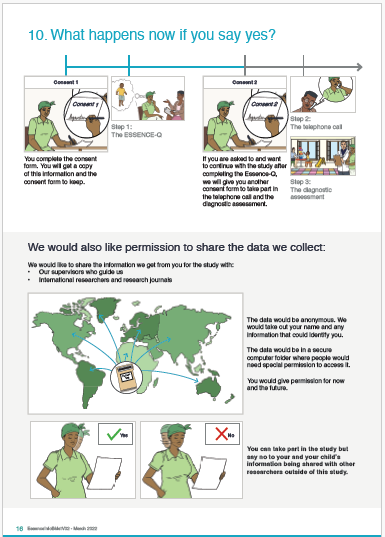
**

**
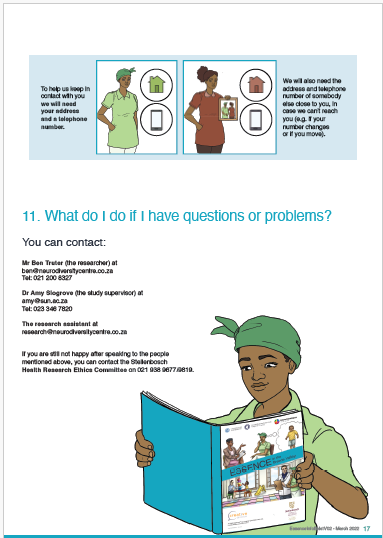
** **
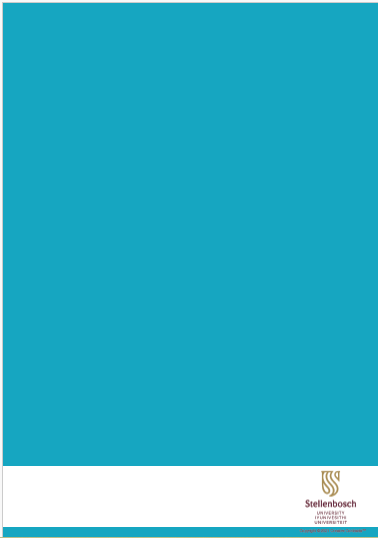
**

**APPENDIX C**

Mother’s feedback rating questionnaire: Afrikaans


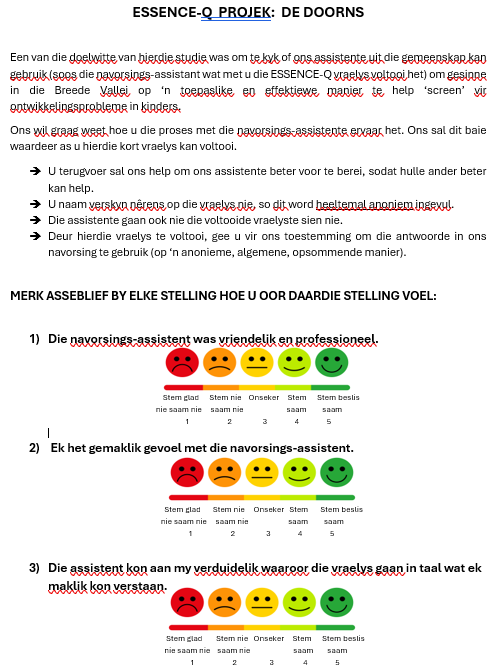


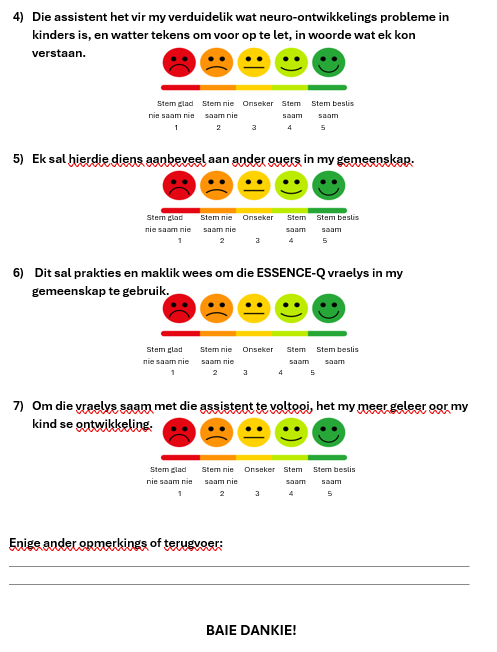

Supplement: Supplementary file 1 — Supplementary Material 1. [file 12888_2025_6791_MOESM1_ESM.docx]
